# Combined levator and frontalis muscle advancement flaps for recurrent severe congenital ptosis

**DOI:** 10.1038/s41433-022-02071-w

**Published:** 2022-04-25

**Authors:** Mostafa Mohammed M. Diab, Khaled Abd-Elaziz, Richard C. Allen

**Affiliations:** 1 Department of Ophthalmology, Faculty of Medicine, Fayoum University, Al Fayoum, Egypt; 2 Department of Ophthalmology, Faculty of Medicine, Beni Suef University, Beni Suef, Egypt; 3 Cullen Eye Institute, Department of Ophthalmology, Baylor College of Medicine, Houston, TX USA

**Keywords:** Eyelid diseases, Surgery

## Abstract

**Background:**

To evaluate the outcomes of combined levator resection and frontalis muscle advancement for surgical management of recurrent severe congenital ptosis.

**Design:**

Retrospective, nonrandomized interventional case series.

**Methods:**

A retrospective review was performed of patients who underwent combined levator resection and frontalis muscle advancement for recurrent congenital ptosis between 2017 and 2020. Inclusion criteria were levator function of 4 mm or less and margin reflex distance 1 (MRD1) of 0 mm or less. Main outcome measures were postoperative MRD 1, lagophthalmos, lash angle, and grades of eyelid contour and crease. The outcomes were assessed by reviewing medical charts and photographs.

**Results:**

Thirty-one patients (35 eyelids) met the inclusion criteria. The mean preoperative MRD1 was −1.14 ± 1.56 mm, which improved to 3.93 ± 0.52 mm with an average lagophthalmos of 0.91 ± 0.74 mm at the last follow-up. A total of 91.4% of eyelids had excellent eyelid contour, crease, and eyelash angle at the final follow-up. One eyelid required revision surgery. There were no other significant complications.

**Conclusions:**

For poor function recurrent congenital ptosis, combining levator resection and frontalis muscle advancement is an effective method that results in long-term correction with cosmetically pleasing outcomes and minimal complications.

## Introduction

Management of poor function and severe congenital ptosis represents a challenging problem. Although numerous surgical techniques have been used, unfavorable functional and esthetic outcomes are not uncommon. In particular, considerable rates of ptosis recurrence have been reported [1–6].

In addition to obstructing the visual axis, severe recurrent ptosis can have a substantial negative psychosocial impact [7, 8]. Reactions of others, particularly in the presence of prominent eyelid and/or forehead scarring from previous surgery, can be stigmatizing. Therefore, repeat surgery is often required [9].

There are few reports on the repair of recurrent poor function ptosis. Repeat frontalis suspension with a new sling [10], sling readjustment [11], and direct frontalis muscle advancement [12] have been attempted. In these techniques, the levator muscle is bypassed and left intact.

Frontalis muscle advancement avoids problems related to graft harvesting and synthetic material implantation. However, this procedure can lead to eyelid malpositions, including anterior advancement of the eyelid away from the globe (eyelid popping), preseptal and pretarsal tenting with crease obliteration, eye lash inversion, and entropion. These problems are assumed to be related to the non-physiologic upward vector of traction of the frontalis flap compared to the normal direction of forces of the levator muscle [6, 13, 14].

In this study, we used levator resection in combination with frontalis flap advancement for repairing recurrent severe ptosis with poor levator function, aiming to make use of its additional lifting power and posterior vector of pull.

## Methods and patients

This is a single-center retrospective case series study of patients who underwent combined trans-eyelid frontalis flap and levator resection for the treatment of recurrent severe congenital ptosis between October 2017 and November 2020. The study was approved by the Scientific Research Ethics Committee at Fayoum University Faculty of Medicine (2020-185). It followed the principles outlined in the Declaration of Helsinki (2008). Written consent was obtained from patients/guardians for the use of patients’ photograph(s) in published media.

The inclusion criteria were patients with recurrent severe ptosis (MRD1 < 1 mm) and absent or poor levator function (≤4 mm) who underwent combined frontalis and levator muscle advancement. Patients with incomplete data or a follow-up period less than 12 months were excluded.

The preoperative data collected included demographics, number of previous ptosis surgeries, type of last surgery, recurrence duration, cause of ptosis recurrence if evident, grading of eyelid and/forehead scarring related to previous ptosis surgery (invisible, visible, and prominent) and preoperative levator function and MRD1.

### Surgical technique

Surgical Technique (Video 1). All surgeries were performed by a single surgeon (MMD) under general endotracheal anesthesia for children and local anesthesia for adults.

**Fig. 1:**
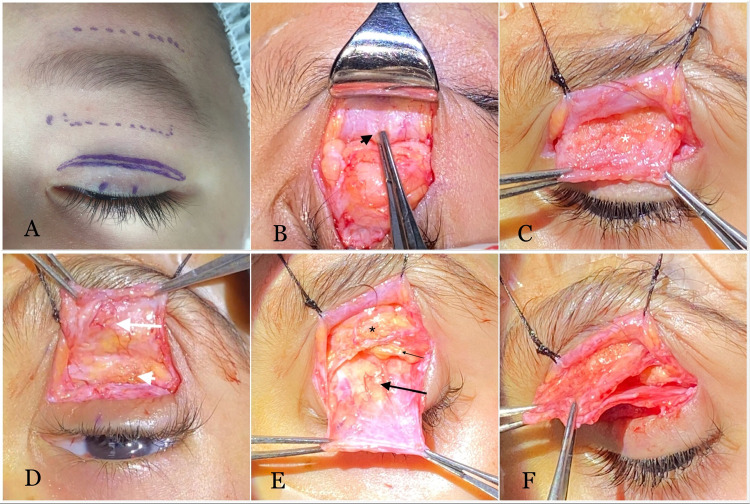
Intraoperative photographs. **A** Skin markings at the eyelid crease and supraoprbital notch. Dotted lines indicate the area of dissection. **B** The deep surface of the junction of the orbicularis and frontalis muscles (black arrow head). **C**, **D** A dissected frontalis flap (white asterisk: anterior surface of frontalis flap; white arrow: deep surface of the frontalis flap; white arrow head: retro-orbicularis oculi fat). **E** A dissected Levator Muller complex flap (black asterisk: retro-orbicularis oculi fat; small black arrow: preaponeurotic fat; large black arrow: levator muscle with prominent fatty infiltration). **F** Double flap.

Preoperative markings were placed at the desired position of the lid crease to be symmetric on both sides and the supraorbital notch (Fig. 1A). The upper lid and brow were infiltrated with 2% lidocaine with 1:100,000 epinephrine, and the face was prepped and draped. An eyelid crease incision was made using a number 15 BD Bard-Parker blade. Dissection was carried out in the suborbicularis plane superficial to the orbital septum and the retroorbicularis oculi fat (ROOF) to ~1 cm above the orbital rim to expose the deep surface of the frontalis-orbicularis oculi muscle. Care should be taken to avoid dissection through the glide plane within the deep galea aponeurotica. The infrabrow segment of the upper lid was everted, and the orbicularis oculi muscle was transected along the inferior brow border (Fig. 1B). Subcutaneous sharp dissection was performed in a prefrontalis plane to ~1 cm above the brow. To improve the mobility of the flap, dissection was continued laterally to the lateral canthus and medially short of the supraorbital notch with or without two short vertical cuts (Fig. 1C, D), and any tethered scar was released. Attention was then directed to the orbital septum, where it was opened to retract the preaponeurotic fat and to expose the levator aponeurosis. The old sling can be dissected and discarded at this point if present. The Müller’s muscle and levator aponeurosis were then dissected from the underlying conjunctiva as a composite flap, and the lateral and medial aspects of the levator aponeurosis were cut with Westcott scissors with caution to avoid injury to the lacrimal gland and the superior oblique muscle tendon. This step allowed adequate mobilization of the flap (Fig. 1E). At this point, two flaps were dissected with good mobility: the frontalis muscle flap and levator-Müller complex flap (Fig. 1F).

A double-armed 6-0 prolene suture was placed at a point 2 mm lateral to the mid-tarsal point through the anterior surface of the tarsal plate ~2 mm below the superior tarsal border. Each arm was passed through both flaps and temporarily tied to check lid height and contour. When the position and contour of the upper eyelid were ideal, the excess tissue of both flaps was excised, leaving a longer end of the upper lid retractor. Two additional sutures were placed medially and laterally to secure the double flap. The skin incision was then closed incorporating the edge of the upper lid retractor for eyelid crease reformation. Frost sutures were used to lift the lower eyelid for corneal protection against exposure.

Postoperative care procedures: antibiotic ointment was applied to the skin wound and the cornea. Eye patches were applied for 24 h, and Frost sutures were removed at 3–5 days postoperatively.

Follow-up examinations were conducted at intervals of 3–7 days, 1 to <2 months, 2–6 months, and 12 months up to each patient’s last follow-up. Photographic documentation was performed. Patients were asked to send a facial photograph with their eyes gently closed every week to evaluate the recovery of safe eye closure function (no corneal exposure).

The postoperative data collected included postoperative MRD1, amount of lagophthalmos on gentle lid closure, postoperative exposure, and eyelid crease and contour grading. Eyelid contour was graded as excellent [3]: natural contour without peaking or flattening; good [2]: mild peaking or flatting, but acceptable; poor [1] eyelid tenting mandating correction. An eyelid crease was graded as excellent [3]: Distinct with a fold; good [2]: distinguishable without a fold; poor [1]: completely obliterated. The eyelash angle was graded as excellent [3]: oriented at 0 to greater than 30°; good [2] inversion but without corneal touch; poor [1] inversion with corneal touch. Patients or their guardians were asked to put a vertical mark on a 100 mm ruler to express their level of satisfaction with the last ptosis surgery (0, lowest: 100, highest). The visual analog scale (VAS) score was recorded on a 100-point scale for each eyelid.

## Results

A total of 31 patients with 35 eyelids were included in this study with a mean follow-up period of 27.32 (SD, 11.23; range, 14–52) months. Among the included patients, 14 (45.2%) were males, and 17 (54.8%) were females. The age ranged from 2 to 23 years, with a mean of 9.23 ± 5.76 years. Eyelid ptosis was unilateral in 27 patients (87.1%) and bilateral in 4 patients (12.9%). Ptosis recurred after levator resection in 5 eyelids (14.3%) and frontalis suspension in 30 eyelids (85.7%) (polypropylene in 11 eyelids, polytetrafluoroethylene (PTFE) in 11 eyelids, and silicone in 8 eyelids). The most common cause of failed frontalis suspension was sling slippage (24/30, 80%). Twenty eyelids (57.2%) had prominent eyelid and/or forehead scarring. The mean preoperative MRD1 in the surgical eyelids was −1.14 ± 1.56 mm, and the mean preoperative levator function was 1.87 ± 1.37 mm. Table 1 details the baseline characteristics.

**Table 1:** Baseline patient characteristics.

		*N* (%)
Age at operation (years)		
Mean (SD)	9.23 (5.76)	
Range	2–23	
Gender, *n* (%)	Male	14 (45.2)
	Female	17 (54.8)
Side, unilateral (%)	Left	13 (41.9)
	Right	14 (45.2)
Type of the last surgery	Polypropylene sling	11 (31.4)
	Expanded polytetrafluoroethylene sling	11 (31.4)
	Silicone sling	8 (22.9)
	Levator resection	5 (14.0)
Technique of failed sling	Closed	11 (42.11)
	Open	8 (33.33)
Cause of failed sling	Explant/infection	5 (14.3)
	Slipped	24 (68.6)
Preoperative levator function, mm, mean (SD)	2.15 (1.47)	
Preoperative MRD1, mm, mean (SD)	−1.14 (1.56)	
Median	−1.00	

All patients showed improvement in MRD1 postoperatively, with an average MRD1 at the last follow-up of 3.93 ± 0.52 mm. Lagophthalmos was observed in all patients postoperatively but gradually decreased with time, with an average of 0.91 ± 0.74 mm at the last follow-up (Supplementary Fig. 1). Safe eyelid closure was achieved in all patients. The average recovery time of safe eyelid closure was 1.87 ± 1.37 weeks.

**Fig. 2:**
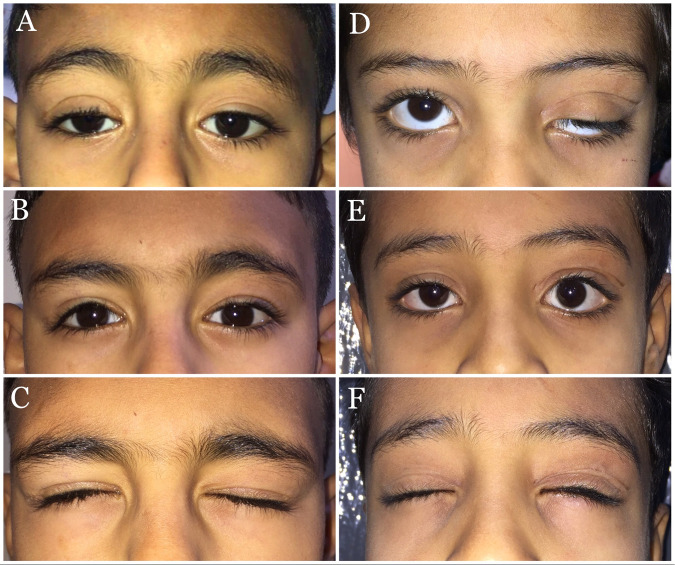
Clinical photographs of two children who underwent combined levator and frontalis muscle advancement. **A** Preoperative photograph of a 6-year-old patient with right upper eyelid recurrent ptosis following levator resection. **B**, **C** Five-year postoperative photographs show good stability of eyelid height, excellent eyelid contour, eyelid crease, and eyelash angle with minimal lagophthalmos. **D** Preoperative photograph of a 9-year-old with left severe ptosis and prominent eyelid scarring following failed levator muscle surgery. **E**, **F** Two-year postoperative pictures show stable eyelid height, excellent eyelid contour, eyelid crease, and eyelash angle without lagophthalmos.

Regarding eyelid contour and crease, 32 eyelids (91.4%) showed excellent outcomes (grade 3) at the last follow-up (Fig. 2; Supplementary Figs. 2, 3, and 4). For the eyelash angle, 22 eyelids (62.9%) had grade 2, 13 eyelids (37.1%) had grade 3 at the first follow-up visit, and 32 eyelids (91.4%) showed grade 3 at the final follow-up visit. The mean subjects’ satisfaction score assessed by VAS was 90.66 (SD, 0.91; range, 70–100). Table 2 demonstrates the postoperative measurements and grades at different follow-up visits.

**Table 2:** Postoperative outcomes at different follow-up visits.

	1^st^ follow-up visit	2^nd^ follow-up visit	3^rd^ follow-up visit	4^th^ follow-up visit
Eyelid crease, *n* (%)				
Distinguishable without a fold	1 (2.9)	1 (2.9)	2 (5.7)	3 (8.6)
Distinguishable with a fold	34 (97.1)	34 (97.1)	33 (94.3)	32 (91.4)
Eyelid contour, *n* (%)				
Mild peaking or flattening but acceptable	9 (25.7)	4 (11.4)	3 (11.4)	3 (8.6)
Natural looking	26 (74.3)	31 (88.6)	31 (88.6)	32 (91.4)
Eyelash angle, *n* (%)				
Inversion without touch	22 (62.9)	6 (17.1)	4 (11.4)	3 (8.6)
Oriented at 0 to greater than 30 degrees	13 (37.1)	29 (82.9)	31 (88.6)	32 (91.4)
Postoperative MRD 1, mm, mean (SD)	5.09 (0.56)	4.49 (0.70)	4.11 (0.53)	3.93 (0.52)
Postoperative lagophthalmos, mm, mean (SD)	2.97 (1.01)	2.06 (1.03)	1.51 (0.95)	0.91 (0.74)
Recovery of safe eyelid closure, *n* (%)				
One week	17 (48.6)			
Two weeks	11 (31.4)			
Three weeks	7 (20.0)			
Visual analog score (SD)	90.66 (6.05)			
Range	(70–100)			
Follow up, month, mean (SD)	27.32 (11.23)			
Range	(14–52)			

**Fig. 3:**
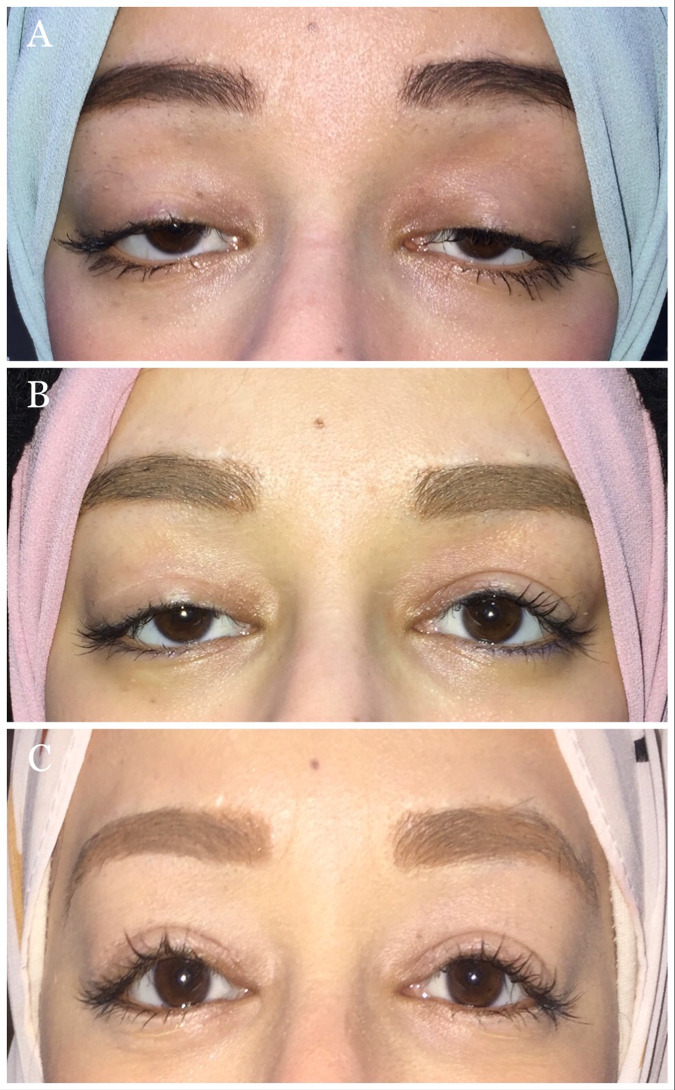
Clinical photographs of a 20-year-old female patient who underwent bilateral combined frontalis and levator muscle advancement. **A** Preoperative picture shows bilateral severe recurrent ptosis after failure of two previous sling procedures. **B** The 3-month postoperative picture shows undercorrection on the right side with satisfactory results on the left side. **C** Three months following revision surgery on the right side.

Twenty eyes had transient mild corneal punctate staining early postoperatively that resolved completely in all patients at the 2^nd^ follow-up (1 to <2 months postoperatively). None of the patients had corneal ulcers. One surgical eyelid showed undercorrection, for which the patient underwent repeat dissection of both flaps with additional advancement (Fig. 3). There were no cases of frontalis muscle paralysis or abnormal eyebrow position or contour.

## Discussion

Considerable rates of ptosis recurrence (7–100%) have been reported following frontalis sling procedures regardless of the material used [1–3, 5]. Similarly, substantial rates of undercorrection have been reported following large levator resections [15]. Medel et al. showed that 50% of cases treated with maximal levator resection needed reoperation [6]. The risk of recurrence increases with time [16] and occurs more frequently and earlier in cases with more severe ptosis [17, 18]. Higher rates of unnatural eyelid contour and crease asymmetry have been reported as well in severe ptosis [19].

In recurrent ptosis, the upper eyelid anatomy may be distorted with tissue scarring from the primary surgery. This makes identification of the eyelid structures and dissection along the anatomical planes challenging. Hemostasis can be difficult due to new vessel formation. Additionally, cicatricial forces may compromise eyelid pliability. These sequelae add to the complexity of severe congenital ptosis surgery [20]. Therefore, repair of recurrent ptosis is more challenging and often has increased operative duration and longer postoperative recovery due to increased edema and ecchymosis. It probably has poorer outcomes than primary surgery.

Sling migration was the most common cause of failed frontalis suspension in the present study. During revision surgery, the sling appeared to have migrated superior to the tarsus. This can be attributed to the effect of the dynamic function of the orbicularis muscle with time in the absence of sufficient bond formation between the implant and the surrounding tissues [21]. This occurred in both open tarsal fixation and closed suspension techniques. Tarsal fixation appears to delay sling migration but does not prevent it. Buttanri et al. [22] proposed that loss of anterior lamellar integrity with the eyelid crease approach can be the reason for recurrence in sutured slings.

Repeat frontalis suspension using a new material has been used by some authors for treating recurrent poor function ptosis. Ural et al. [10] performed 20 repeat frontalis suspension procedures for recurrent ptosis of various severities with a success rate of 65%. The success was directly related to the levator function and the severity of ptosis. However, there was no comment on the type of sling material. Buttanri et al. [22] performed 13 revision surgeries for failed silicone slings by reattaching the same sling to the tarsal plate or replacing it with a new sling. Alloplastic materials are known to have high recurrence rates with the possibility of sling cheese wiring, extrusion, and infection [3, 23]. Therefore, it is believed that reintroduction of another alloplastic sling should be avoided due to its inherent hazards following a previously failed material [2].

Autogenous fascia lata has been considered the standard material given its relatively durable results and minimum risk of extrusion. However, permanent thigh scars and other donor site-related complications can be problematic [24]. Additionally, some studies showed similar results with autogenous and synthetic materials [2, 3]. One study with a long-term follow-up demonstrated increasing rates of cosmetic deterioration, including poor eyelid crease and lash inversion, with time among children treated with fascia lata suspension [25].

To avoid harvesting a new graft, Lee et al. [11] dissected and reattached the pre-existing fascia lata to the tarsal plate in cases of undercorrected ptosis. However, identification of the implanted fascia can be difficult as it often becomes integrated into the surrounding tissues with scar tissue formation with increased risk of its damage or it can undergo absorption with time.

Assuming that traditional ptosis correction techniques are unreliable in patients with previous eyelid surgery due to distorted anatomy, Bassin et al. [26] described a full-thickness eyelid resection for residual ptosis. Similarly, Codner and McCord [27] performed en bloc resection of a portion of the posterior lamellar for ptosis with scarred eyelids.

Conventional frontalis flap advancement techniques can have an increased risk of prolonged lagophthalmos following surgery [28] and undercorrection [6, 29, 30]. Several unfavorable cosmetic outcomes have been reported as well, including eyelid crease obliteration, abnormal eyelid contour with angulation deformities, poor eyelid apposition to the globe, lash inversion, and entropion [6, 28]. This is likely due to the abnormal direction of force.

The procedure described in the current study attempts to address many of the issues associated with reoperation in recurrent congenital ptosis. By including the levator complex, additional elevation is achieved. In severe congenital ptosis, levator muscle function is often markedly decreased or absent. Therefore, levator muscle surgery alone may not be sufficient to provide effective or durable eyelid lifting [31]. Additionally, large resections are often required, which may result in a frozen eyelid with an increased risk of exposure keratopathy. However, in the presence of the elevating power of the frontalis, the additional levator function, although weak, may produce an augmenting effect without the need for very large resections. Furthermore, Eton et al. assumed that the levator muscle, even when dystrophic, is vital to activate the natural neurologic circuitry to clear the visual axis [32].

By using two flaps, the traction forces of the frontalis muscle may be redirected. The force exerted by the frontalis muscle for elevating the lid tends to pull the lid away from the globe (eyelid popping), obliteration of the eyelid crease, and preseptal tenting with an unnatural appearing eyelid [33]. Concurrent levator advancement modifies the traction forces into a more physiologic direction, enhancing the horizontal vector and negating the undesirable effects of the vertical vector. It aids in maintaining lid apposition to the globe, particularly in upgaze and with brow elevation. Vasquez et al. [34] used a levator aponeurosis pulley to redirect the vertical pull of FMF into a more horizontal vector to avoid poor lid positioning. However, this may weaken an already dystrophic muscle.

Entropion and/or eyelash inversion are known shortcomings to direct frontalis advancement. This can be attributed to the marked posterior lamellar tightening by frontalis advancement with subsequent relative anterior lamellar laxity in addition to the non-physiologic vertical vector of pulling forces [29]. To avoid this complication, eyelid crease reformation sutures are performed (skin and muscle of the inferior wound edge-levator Muller flap-skin of the superior wound edge) in all cases. These sutures help restore lamellar balance by applying a posterior vector of pull on the anterior lamella with effective eyelash eversion.

Last, all patients who underwent combined flap reconstruction achieved safe eyelid protective closure within 1–3 weeks following surgery. This can be attributed to the following factors: first, the frontalis flap itself is a dynamic structure having internal contractile force with good elasticity. Therefore, eyelid closure is enhanced by relaxation of the frontalis muscle and eyebrow descent [35]. Second, good mobility of both flaps is ensured before fixing them to the tarsus. Third, the palpebral orbicularis muscle is preserved [36] to ensure good eyelid closing force.

In contrast to our results, Hao et al. [20] reported that the average recovery time of safe eyelid closure was 3.9 ± 1.04 months following CFS suspension for recurrent ptosis. This relatively long period can be explained by the assumption that CFS is a fixed suspension system.

In the present study, one patient experienced crease obliteration with undercorrection on one side following bilateral correction. A revision surgery was performed, entailing resecting some of the double flap and advancing it further. There was no need for extended dissection. This demonstrates that this technique is repeatable.

Limitations of the present study include the retrospective design and small cohort of patients. Ideally, a randomized, prospective study comparing frontalis muscle advancement flap alone to the described procedure would help determine if the addition of the levator-Muller muscle flap results in a significant difference.

In conclusion, frontalis muscle advancement provides effective eyelid elevation in recurrent severe ptosis. Adjuvant levator-Müller complex advancement appears to maximize the physiologic synergistic action of the frontalis and the levator muscles and enhance functional and cosmetic outcomes.

### Summary

#### What was known before

- Management of severe poor-function congenital ptosis continues to be controversial, with high rates of unfavorable outcomes.
- Direct frontalis flap advancement represents an effective method of treating primary severe ptosis while avoiding complications related to sling materials and graft harvesting.
- Frontalis flap advancement can lead to eyelid malpositions due to the nonphysiologic upward vector of traction of the flap.

#### What this study adds

- Direct frontalis flap advancement is reliable for the treatment of recurrent severe ptosis.
- Adjuvant levator muscle advancement appears to maximize functional and cosmetic outcomes. It helps prevent eyelid malpositions associated with frontalis flap advancement, including eyelash inversion, entropion, and eyelid popping.

## Supplementary information

Supplementary figure 1

Supplementary figure 2

Supplementary figure 3

Supplementary figure 4

Supplementary figure legends

Combined maximal levator resection anf frontalis flap advancement for treating recurrent severe ptosis.

## Data Availability

The data that support the findings of this study are available from the corresponding author with the permission of Fayoum University Hospital upon reasonable request.
