# Supplementary figures and images for: Combined levator and frontalis muscle advancement flaps for recurrent severe congenital ptosis
Source: Eye (Lond). 2022 Apr 25;37(6):1100–6. doi: 10.1038/s41433-022-02071-w (PMC10102021; doi:10.1038/s41433-022-02071-w)

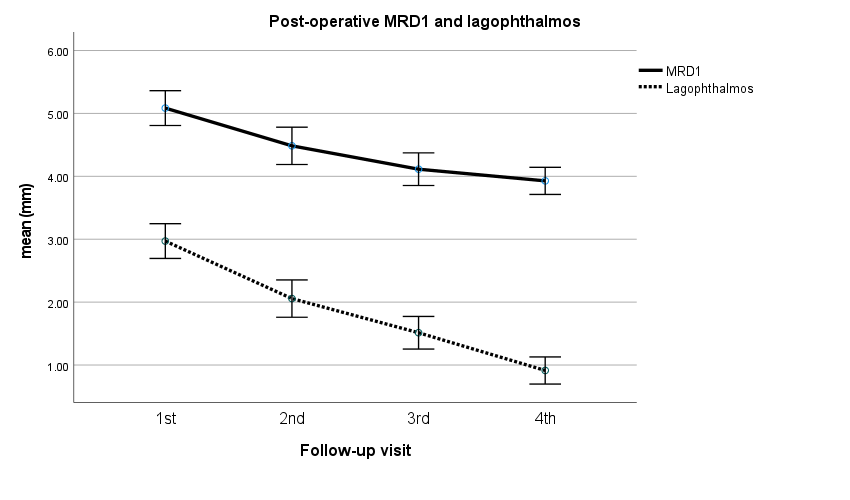

Supplement: Supplementary file 1 — Supplementary figure 1 [file 41433_2022_2071_MOESM1_ESM.png]

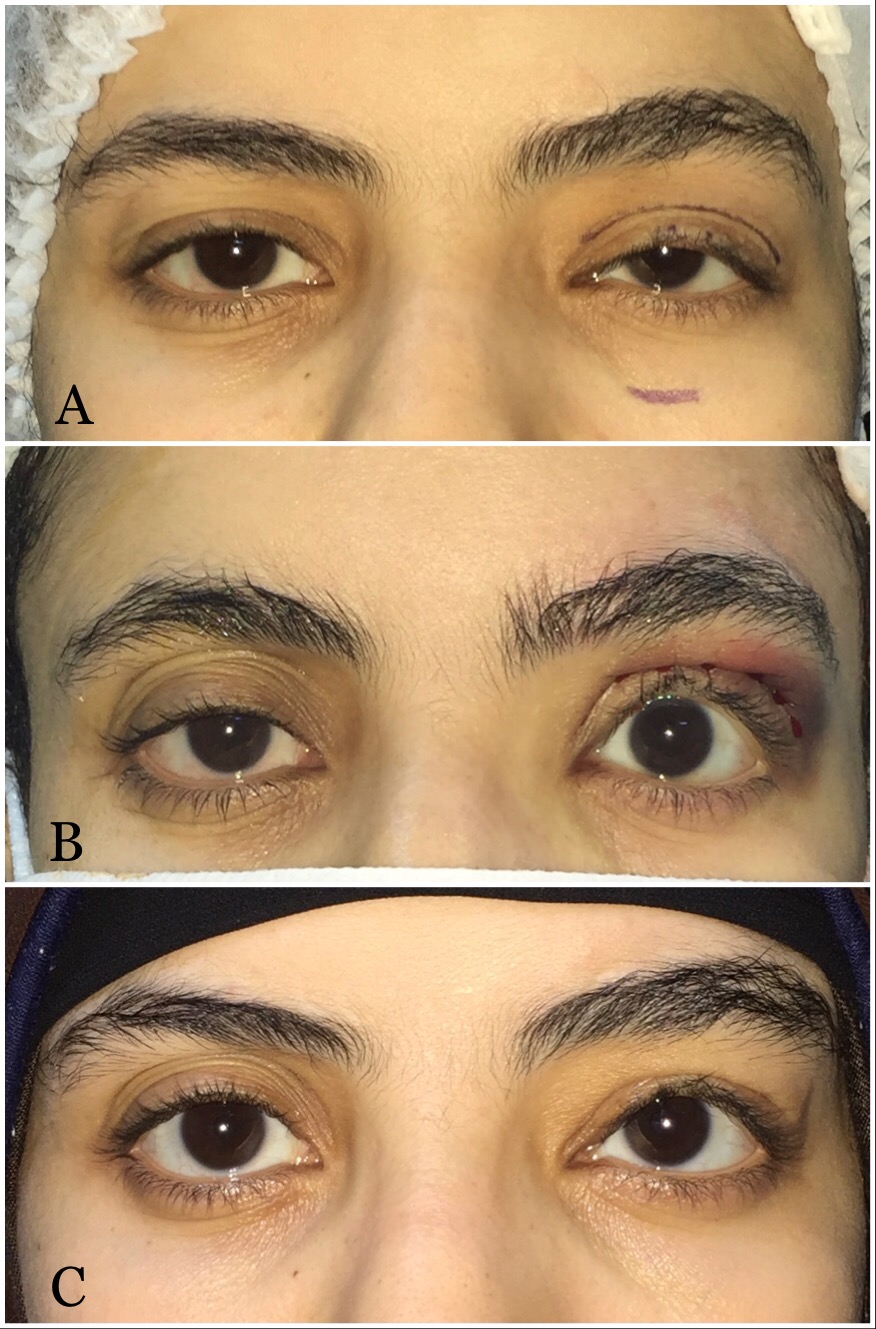

Supplement: Supplementary file 2 — Supplementary figure 2 [file 41433_2022_2071_MOESM2_ESM.jpg]

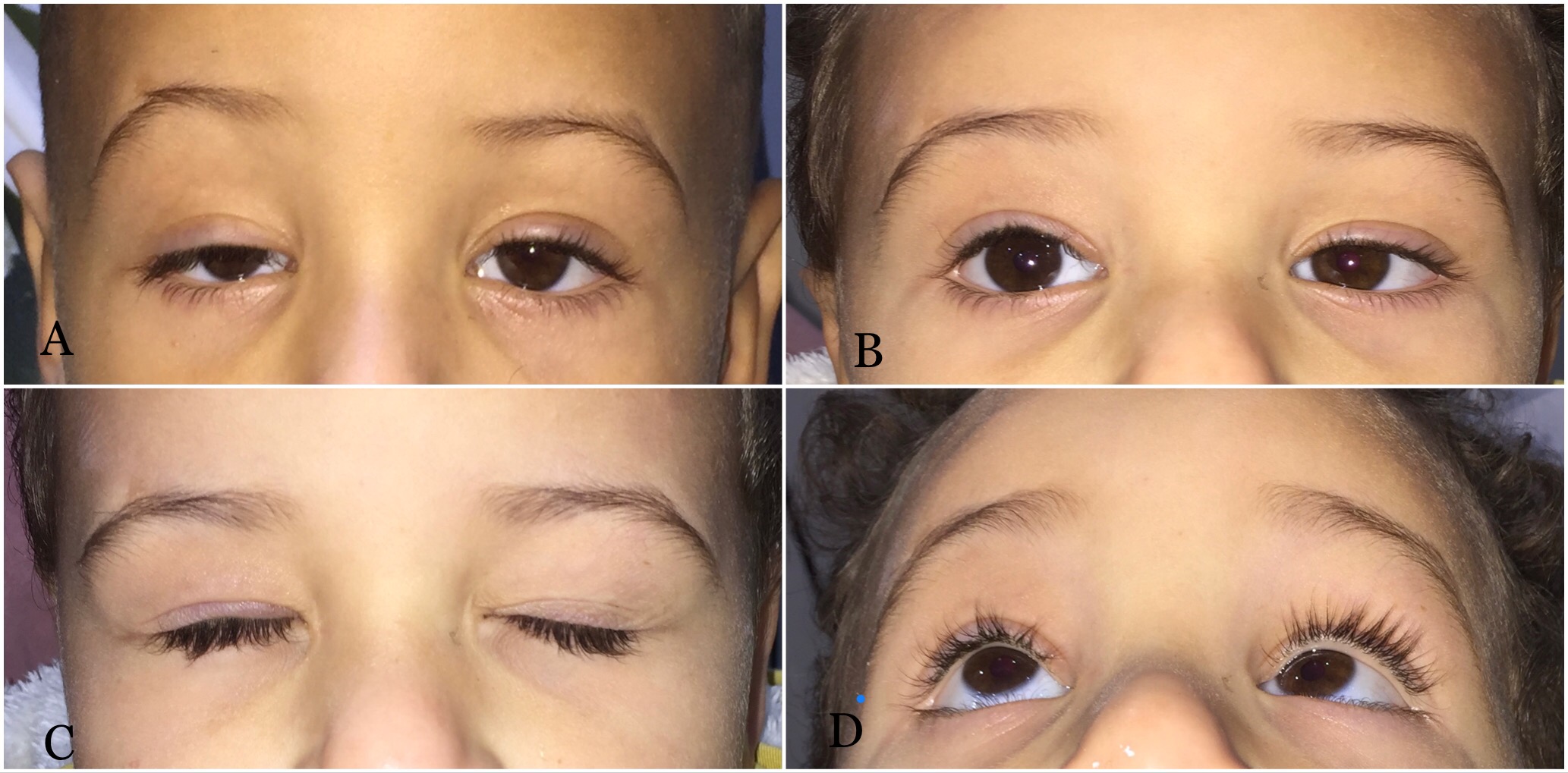

Supplement: Supplementary file 3 — Supplementary figure 3 [file 41433_2022_2071_MOESM3_ESM.jpg]

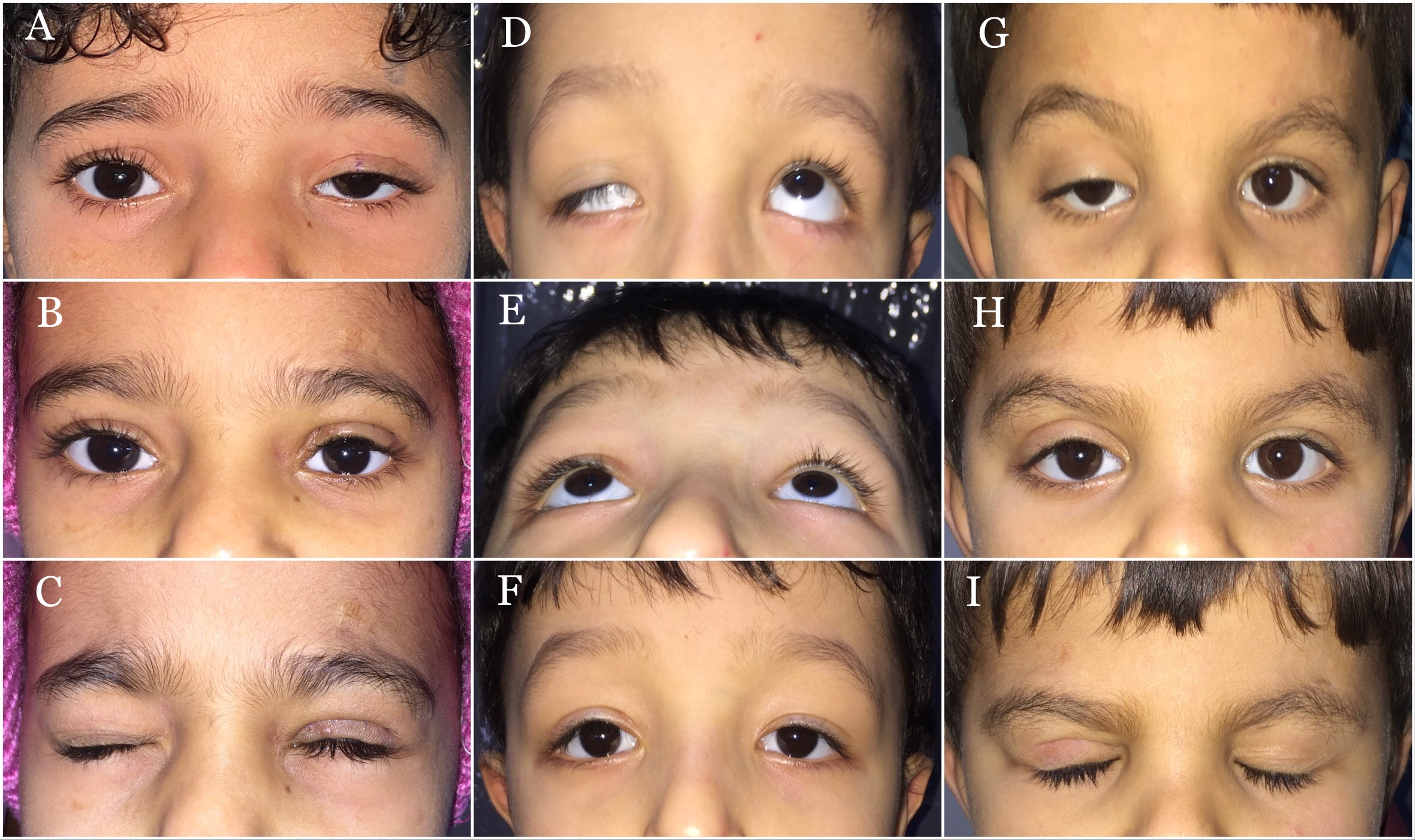

Supplement: Supplementary file 4 — Supplementary figure 4 [file 41433_2022_2071_MOESM4_ESM.jpg]
