# Supplementary material for: Combined levator and frontalis muscle advancement flaps for recurrent severe congenital ptosis
Source: Eye (Lond). 2022 Apr 25;37(6):1100–6. doi: 10.1038/s41433-022-02071-w (PMC10102021; doi:10.1038/s41433-022-02071-w)
Supplement: Supplementary file 5 — Supplementary figure legends [file 41433_2022_2071_MOESM5_ESM.docx]

Supplementary figure 1. Postoperative mean margin reflex distance 1 and lagophthalmos.

Supplementary figure 2. Clinical photographs of a 23-year-old female patient having recurrent ptosis of the left upper eyelid. She underwent three procedures to correct ptosis in her childhood. A, Preoperative appearance. B, Immediate postoperative appearance after combined levator and frontalis advancement showing limited edema. C, 2 years postoperation, improved eyelid height with good stability.

Supplementary figure 3. Clinical photographs of a 6-year-old child with recurrent ptosis of the right upper eyelid following silicone sling. A, Preoperative appearance. B-D, one year postoperation, good eyelid height in primary position, 0-1 mm lagophthalmos, and excellent lash eversion with good lid apposition to the globe in upgaze.

Supplementary figure 4. Clinical photographs of three different children with recurrent severe ptosis following failed slings. Preoperative appearance (A, D, and G). B and C, one week postoperation, good eyelid position with minimal edema in primary gaze and the operated eye can be safely closed. E and F, one year postoperation, effective eyelid elevation in upgaze with good apposition to the globe and excellent symmetry in primary position. H and I, six months postoperation, effective eyelid elevation and closure.
